# Streamlining heterologous expression of top carbonic anhydrases in *Escherichia coli:* bioinformatic and experimental approaches

**DOI:** 10.1186/s12934-024-02463-5

**Published:** 2024-07-02

**Authors:** Hui Wei, Vladimir V. Lunin, Markus Alahuhta, Michael E. Himmel, Shu Huang, Yannick J. Bomble, Min Zhang

**Affiliations:** 1 Biosciences Center, National Renewable Energy Laboratory, Golden, CO 80401 USA; 2 National Bioenergy Center, National Renewable Energy Laboratory, Golden, CO 80401 USA; 3 Present Address: Thayer School of Engineering, Dartmouth College, Hanover, New Hampshire USA

**Keywords:** Carbonic anhydrase, Protein expression, Protein solubility prediction, Phylogenetic tree analysis, *Escherichia coli*

## Abstract

**Background:**

Carbonic anhydrase (CA) enzymes facilitate the reversible hydration of CO_2_ to bicarbonate ions and protons. Identifying efficient and robust CAs and expressing them in model host cells, such as *Escherichia coli*, enables more efficient engineering of these enzymes for industrial CO_2_ capture. However, expression of CAs in *E. coli* is challenging due to the possible formation of insoluble protein aggregates, or inclusion bodies. This makes the production of soluble and active CA protein a prerequisite for downstream applications.

**Results:**

In this study, we streamlined the process of CA expression by selecting seven top CA candidates and used two bioinformatic tools to predict their solubility for expression in *E*. *coli*. The prediction results place these enzymes in two categories: low and high solubility. Our expression of high solubility score CAs (namely CA5-SspCA, CA6-SazCAtrunc, CA7-PabCA and CA8-PhoCA) led to significantly higher protein yields (5 to 75 mg purified protein per liter) in flask cultures, indicating a strong correlation between the solubility prediction score and protein expression yields. Furthermore, phylogenetic tree analysis demonstrated CA class-specific clustering patterns for protein solubility and production yields. Unexpectedly, we also found that the unique N-terminal, 11-amino acid segment found after the signal sequence (not present in its homologs), was essential for CA6-SazCA activity.

**Conclusions:**

Overall, this work demonstrated that protein solubility prediction, phylogenetic tree analysis, and experimental validation are potent tools for identifying top CA candidates and then producing soluble, active forms of these enzymes in *E. coli*. The comprehensive approaches we report here should be extendable to the expression of other heterogeneous proteins in *E. coli*.

**Supplementary Information:**

The online version contains supplementary material available at 10.1186/s12934-024-02463-5.

## Background

Carbonic anhydrase (CA, EC 4.2.1.1) is a family of metalloenzymes that catalyzes the reversible hydration of carbon dioxide (CO_2_) to bicarbonate ion (HCO_3_^−^) and proton (H^+^). CAs from different species are divided into five evolutionarily distinct classes, the α-, β-, γ-, δ- and ζ-classes [1–3]. They play important roles in regulating pH, CO_2_ transport, and biomineralization in various biological processes. Recent years have seen an increased interest in using these enzymes for CO_2_ capture, utilization, and storage (CCUS) processes [4, 5].

Two features of CAs are key for downstream applications: thermostability and specific performance. Thermostable CAs are enzymes that can withstand high temperatures without losing their catalytic activity. Examples of promising thermostable CAs include: SspCA from *Sulfurihydrogenibium yellowstonense* YO3AOP1 which withstands temperatures up to 100 °C [6, 7] and SazCA (*Sulfurihydrogenibium azorense*) which is stable at temperatures up to 95 °C [8, 9]. These thermostable CAs have potential applications in biotechnology, especially in industrial processes that require high temperatures.

High specific activity CAs are useful for carbon dioxide capture and sequestration, biocatalysis, and biomedical imaging. The most catalytically efficient CA reported to date is SazCA from *S. azorense*, with turnover number *k*_*cat*_ of 4.4 × 10^6^ s^−1^ [8, 10], a *k*_cat_/*K*_M_ value of 3.5 × 10^8^ M^−1^ s^−1^ [10]. SspCA from *S. yellowstonense* YO3AOP1 also showed a good catalytic activity, with a *k*_*cat*_ value of 9.35 × 10^5^ s^−1^ and a *k*_*cat*_*/K*_*M*_ value of 1.1 × 10^8^ M^−1^ s^−1^ [11].

The potential for the utilization of CAs is limited not only by the harsh conditions required in these processes, but also by the high cost for producing these enzymes at large scale. Overexpressing CA in *Escherichia coli* has been widely used for producing large quantities of enzymes for various applications, including biological research, pilot plant testing, and industrial processes. However, overexpressing proteins in *E. coli* can often lead to the formation of insoluble protein aggregates, or inclusion bodies. The production of soluble and active protein is a prerequisite for downstream applications.

Recently, computational methods have been developed to provide tools to address the obstacles historically posed for effective heterologous protein expression in *E. coli*. These methods predict protein solubility from their amino acid sequences [12–14]. Since protein solubility and activity are often correlated [14], such tools can be extended to indirectly predicting the activity of proteins. However, such tools developed by different groups used different heterogeneous databases, and thus the experimental details of these databases are often inconsistent and without proper documentation [15].^.^

The long-term goal of this study is to develop a novel biological, low energy-consuming, and sustainable CO_2_ waste gas scrubbing technology. We aim to develop this technology using the following approaches.

**(1)** Improving the robustness of CA enzymes, including tolerance to high pH, high solvent concentration, and high temperature. We began with conducting a detailed literature search to select candidate CAs from diversified sources (i.e., bacteria isolated from various habitats). One issue we have encountered with the published CA literature is the challenge in comparing thermal tolerance and kinetics data from different research groups. It is clear that the same conditions must be used for the expression, purification, and testing of these enzymes.

**(2)** Using protein solubility prediction programs to guide the selection of candidate CAs and increase success rate for their overexpression in *E. coli*. To avoid the potential prediction biases from using a single tool, this study used two of the frequently used tools based on amino acid sequence for predicting the solubility of CA expression in *E*. *coli*. In total, seven CA candidates were selected and predicted to be soluble when expressed in *E. coli*. After expression in *E. coli*, the CA enzymes were purified by column chromatography, followed by enzymatic characterization. Four CAs were found to have a high production and purification yield (in the range of 5 to 75 mg L^−1^ in flask culture), including the most thermostable CA5-SspCA and the most effective CA6-SazCA’s variant CA6-SazCAtrunc.

## Materials and methods

### Calculation for the pI and MW of CA proteins

The pI and molecular weight (MW) of CAs were calculated using the ExPASy Compute pI/Mw tool developed by the Swiss Institute of Bioinformatics (https://web.expasy.org/compute_pi/). The protein sequences used for calculations included 6 × His-tags.

### Solubility prediction for CA overexpression in *E. coli*

The solubility prediction of CA proteins was determined using two web tools: SOLpro (https://scratch.proteomics.ics.uci.edu/) [16], and Protein-Sol (https://protein-sol.manchester.ac.uk) [17]. The interpretations for the prediction scores generated by these web servers are: (1) In Protein-Sol method, if the solubility of the protein (QuerySol) was greater than the average of protein population (PopAvrSol = 0.45), it confirms the soluble nature of the proposed protein [17]; and (2) In SOLpro method, the solubility is predicted by assigning the probability scores, where soluble proteins receive scores of  ≥ 0.5, whereas insoluble proteins receive scores of  < 0.5 [16].

### CA construct design

We have designed two types of CA-expressing constructs based on the eight constructs we have built and tested (Table 1). Most of these CA candidates were selected from α-class CAs because of their high thermostability and/or high catalytic efficiency, as reported in literature listed in Table 1. In addition, two γ-class CAs (i.e. CA7 and CA8) were also selected from hyperthermophilic archaea species with presumable or experimentally confirmed thermostability [18, 19]. More detailed descriptions were provided in the protein purification sections for each individual CAs. The default placement of the 6 × His-tag was on the C-terminus of the construct, but in one case where we suspected that the His-tag would interfere with the protein multimerization, the 6 × His-tag was placed on the N-terminus.

**Fig. 1:**
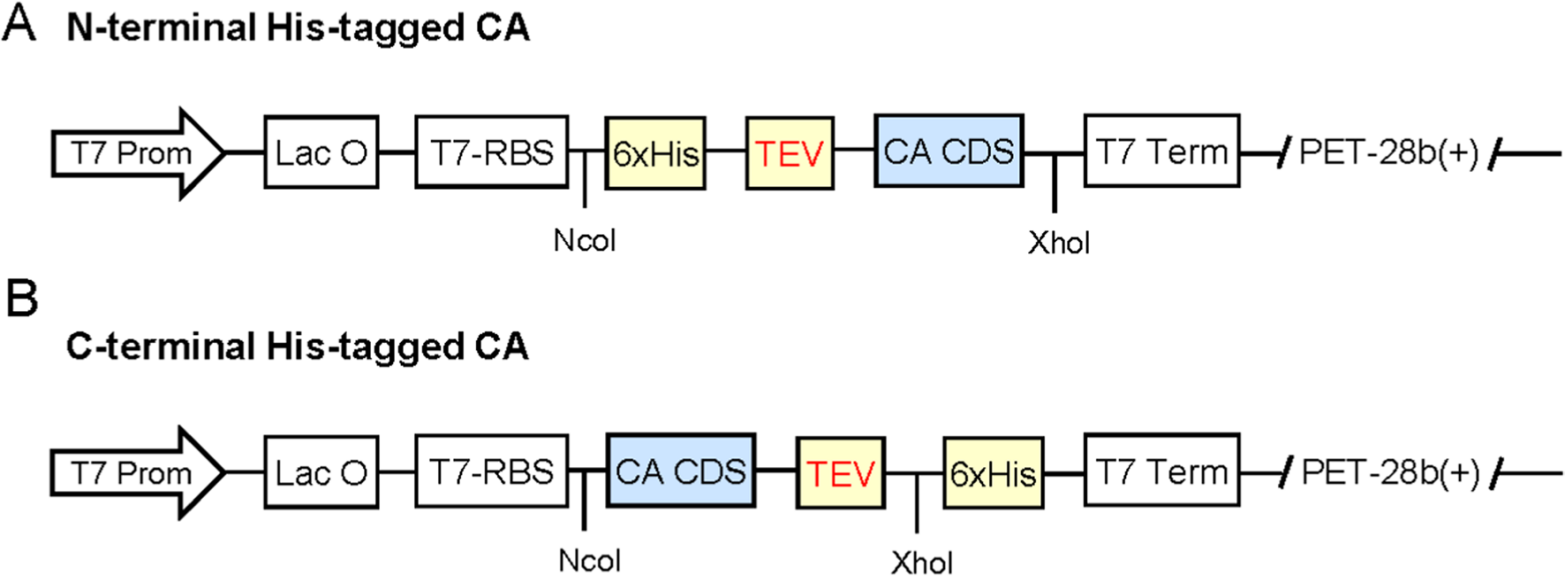
Plasmid construction for N-terminal (**A**) and C-terminal (**B**) His-tagged CAs. It involved cloning the synthetic genes into pET-28( +) between the NcoI and XhoI restriction sites. The expression of CA proteins was driven by the T7 promoter (T7 Prom) and terminated by the T7 terminator (T7 Term). Lac O denotes the Lac operator, and RBS indicates the ribosome binding site

- *(1) Type I: N-terminal His-tagged CAs*  One CA, namely TaCA (*Themovibrio ammonificans* CA), was selected and the corresponding plasmid was designed for expression with N-terminal His-tag. The design for HisTag-TEV-CA was illustrated in Fig. 1A and briefly described as below. The synthesized gene had NcoI site (ccatgg) plus two nucleotides of guanines (i.e. ccatgggg; which contains a start codon as underlined) at 5′ end of synthesized fragment (to make it in-frame with the rest of cDNA), followed by (a) the 6 × His (HHHHHH) and the TEV site (ENLYFQG), and (b) the catalytic domain sequence of specific CA. The synthesized gene was codon-optimized using *E. coli* codon usage frequency and synthesized by GenScript Inc. The synthesized gene was digested with NcoI-XhoI, and linked into NcoI-XhoI digested PET-28b( +) vector.
- *(2) Type II: C-terminal His-tagged CAs  *Seven CAs have been selected and designed for expression with C-terminal His-tag, as listed below in alphabetic order of species names for the CA sources: LOGACA from deep sea thermal vent; PabCA from *Pyrococcus abyssi;* PhoCA from *Pyrococcus horikoshii*; PmaCA from *Persephonella marina*; SazCA from *Sulfurihydrogenibium azorense*; SspCA from *Sulfurihydrogenibium *sp. (i.e. *Sulfurihydrogenibium yellowstonense*) strain YO3AOP1; TaCA from *T. ammonificans.*
  The design for CA-TEV-HisTag was illustrated in Fig. 1B and described as follows. The synthesized genes had NcoI site (ccatgg) plus two nucleotides (gc) (i.e. ccatgggc) at 5’ of synthesized fragment (to make it in-frame fit with the rest of cDNA and the C-terminal 6xHis-tag), followed by (a) the catalytic domain sequence of specific CA, and (b) the TEV site (ENLYFQG). The gene sequences were codon-optimized using *E. coli* codon usage frequency and synthesized by GenScript Inc for each of the seven constructs. The synthesized gene fragment was digested with restriction enzymes of NcoI and XhoI, and linked into NcoI-XhoI digested PET-28b( +) vector.

**Table 1:** Plasmids designed and constructed for intracellular expression of carbonic anhydrase (CA) enzymes in *E. coli* strain BL21-CodonPlus (DE3)-RIL

Strain	Plasmids	Source, amino acid no., pI, MW, and reasons for being selected	CA classes
Type I: CAs with N-terminal His-tag			
*E*. *coli* CA1	Gene1-pET-28b-HisTag-TEV-TaCA	*T. ammonificans*; 240 aa; pI 8.96; MW 27.5 kDa One of most thermostable CAs [4], but poorly expressed in *E. coli* [20]	α-class
Type II: CAs with C-terminal His-tag			
*E*. *coli* CA2	Gene2-pET-28b-TaCA-TEV-HisTag	*T. ammonificans*; 240 aa; pI 8.96; MW 27.5 kDa	α-class
*E*. *coli* CA3	Gene3-pET-28b-PmaCA-TEV-HisTag	*P. marina*; 237 aa; pI 6.86; MW 27.4 kDa; PBD: 6IM0_1 One of most thermostable CAs [4]	α-class
*E*. *coli* CA4	Gene4-pET-28b-LOGACA-TEV-HisTag	Metagenomic source; 239 aa; pI 7.93; MW 27.3 kDa Highly thermostable at alkaline pH [21]	α-class
*E*. *coli* CA5	Gene5-pET-28b-SspCA-TEV-HisTag	*S. yellowstonense* YO3AOP1; 239 aa; pI 9.04; MW 27.8 kDa; PDB: 4G7A_A One of most thermostable [4] and efficient CAs [11]	α-class
*E*. *coli* CA6trunc	Gene6-pET-28b-SazCA-TEV-HisTag	*Sulfurihydrogenibium azorense*; 239 aa; pI 7.92; MW 27.7 kDa; PDB: 4X5S_A One of most thermostable [4] and efficient CAs [10]	α-class
*E*. *coli* CA7	Gene7-pET-28b-PabCA-TEV-HisTag	*Py. abyssi*; 185 aa; pI 6.36; MW 20.4 kDa Representative of γ-class CA	γ-class
*E*. *coli* CA8	Gene8-pET-28b-PhoCA-TEV-HisTag	*Py. horikoshii*; 185 aa; pI 6.22; MW 20.5 kDa Thermostable CA [18, 19], as a comparison with α-class CA	γ-class

The MW of the catalytic domain of CAs, as described in the table, did not include the His-tag (HHHHHH) and tobacco etch virus (TEV) protease recognition site (ENLYFQG); thus, the MW of expressed CA = listed MW of catalytic domain + MW of His-tag (0.84 kDa) + MW of TEV site (0.87 kDa). *CA* carbonic anhydrase, *MW* molecular weight

### Transformation

Each of the above eight pET-28b-serial plasmids carrying CA genes were transformed into BL21-CodonPlus (DE3)-RIL *E. coli* competent cells (Cat. 230,245, Agilent Technologies), and positive single colonies were picked from selection plates containing kanamycin (Kan; 50 µg/mL) and chloramphenicol (CAM; 30 µg/mL).

### Medium supplementation and cell culture

CA protein-expressing transformants were cultured in LB supplemented with both 50 µg/mL kanamycin and 30 µg/mL chloramphenicol and induced with IPTG for the CA enzyme expression. For seed culture, cells were grown aerobically in the above expression medium under 37 C and 225 rpm overnight.

For large-scale culture (1- to 2-L) of His-tagged CAs, 20 mL seed culture were inoculated into 1 L of the above medium in a 2-L flask; in total, 2 L culture was needed to get appropriate amount of cell pellets for downstream protein extraction. The culture was grown aerobically under 37 °C and 225 rpm until OD600 reached a value of 0.8. The culture was placed on ice for 3 h, followed by adding 0.5 mM ZnSO_4_ and 0.5 mM IPTG (final concentration in the medium) as the optimized induction condition. The cooled culture was incubated overnight at 20 °C and 225 rpm.

To collect cell samples for CA expression level analysis, a 2 mL broth sample was taken before IPTG induction (at lower cell density) and a 1 mL broth (at higher cell density) sample was taken after overnight IPTG induction. Cells were collected by micro-centrifugation for 60 s at 4 °C, washed with sterilized/distilled water, and then pelleted by centrifugation. The cell pellets were flash-frozen in liquid nitrogen and transferred to a −80 °C freezer until used for protein extraction.

### Cell lysis, protein extraction, and SDS-PAGE analysis for CA induction analysis

The extraction of soluble protein was performed following a procedure modified from literature [22, 23]. The above-collected and washed cell pellets were suspended in 90 µL of 10 mM Tris HCl buffer (pH 7.0) in Eppendorf tubes. Cells were lysed by sonication in ice (Cole-Parmer, model EW-08895-59) using 40 kHz pulses for 5 min (10 s on and 10 s off). The lysed cell suspensions were centrifuged at 12,000x*g *for 2 min at 4 °C and the supernatant fractions were collected and designated as soluble proteins. Protein concentrations were measured at 280 nm using a NanoDrop UV–Vis Spectrophotometer (Thermo Fisher Scientific, Waltham, MA, USA). The cleared lysate was further processed by adding 30 µL 4 × Laemmli sample buffer, incubated using a hot block at 70 °C for 10 min, and designated as soluble proteins. The cell pellets were resuspended in 120 µL 1 × Laemmli sample buffer, boiled for 5 min, and designated as insoluble proteins. All samples were stored at −80 °C until further analysis. The soluble and insoluble fractions were analyzed using SDS-PAGE [NuPAGE Novex 4–12% Bis–Tris Mini Gel (Invitrogen, Thermo Fisher Scientific, Waltham, MA)] in NuPAGE MES buffer with SeeBlue Plus2 Prestained Protein Standards (LC5925; Invitrogen, NY) as the MW markers.

### Protein purification

CA proteins that expressed in *E. coli* with C- or N-terminal 6xHis-tags were purified from cell lysates using affinity chromatography followed by size-exclusion chromatography (SEC) with an Akta Pure chromatography system (Cytiva, Marlborough, MA, USA). HisTrap FF column (5 mL, GE Healthcare) or Cobalt His-Pur column (Thermo Scientific, Rockford, IL) was equilibrated with 20 mM Tris pH 7.5, 150 mM NaCl and 10 mM imidazole, cell lysate was loaded on the column using a 20 mL loop, and after washing the column with equilibration buffer protein sample was eluted using 20 mM Tris pH 7.5, 150 mM NaCl and 250 mM imidazole. Elution fractions with protein (absorbance at 280 nm) were concentrated together and loaded on a Superdex 75 (26/60) size exclusion chromatography column (GE Healthcare, Piscataway, New Jersey, USA) with 20 mM Tris pH 7.5, 150 mM NaCl. The resulting fractions with protein (absorbance at 280 nm) were collected and analyzed using SDS-PAGE [NuPAGE Novex 4–12% Bis–Tris Mini Gel (Invitrogen, Thermo Fisher Scientific, Waltham, MA)] for purified CAs.

### Activity measurement

CA activity was measured as close to 0 °C as possible in an ice-water bath via Wilbur-Anderson method [24] by direct pH measurement taken with 1 s intervals. Ten millilitre of 20 mM Tris buffer with pH 8.3 was mixed with 10 mL 50% saturated CO_2_ solution. Time required for pH to change from 8.0 to 6.3 was measured. For catalyzed reaction 10 µL of 0.1 mg/mL CA was added to the buffer prior to CO_2_ solution addition.

Activity in WAU (Wilbur-Anderson Units) was calculated as 1 WAU = (T−T^c^)/T^c^ where T is time for uncatalyzed reaction in seconds and T^c^ is time for catalyzed reaction in seconds.

## Results and discussion

### Literature analysis for the selection of top CAs

A comprehensive literature analysis was conducted to identify the top CAs with regard to specific activity and thermostability. Each of these CAs are listed in Table 2 and highlighted below with numeric IDs (i.e., CA1-CA8) to streamline the subsequent expression and phylogenetic analyses. Previously, these different CAs were expressed by different groups using different methods and conditions. Among them, CA3-PmaCA has been previously expressed in *E. coli* with N-terminal His-tag [25]. PmaCA enzyme from this organism without a signal peptide was expressed in *E. coli* at levels five-fold higher than that with signal peptide [26]. CA5-SspCA was expressed with N-terminal His-tag in *E. coli* and purified by His-select HF Nickel affinity gel to a 95% purity [6]. Similarly, CA6-SazCA was expressed with N-terminal His-tag in *E. coli* [8–10]. However, CA8-PhoCA protein was expressed in *E. coli* with C-terminal His-tag [19].

**Table 2:** Solubility prediction using the Protein-Sol and SOLpro programs for the expression of top selected CA proteins in *E. coli*

CA classes	CA IDs	(1) Protein-Sol score [17]	(2) SOLpro soluble probability [16]	Average solubility score
CAs with low solubility score				
α-class	CA4; LOGACA-His	0.44	0.72	0.58
α-class	CA1; His-TaCA	0.47	0.72	0.60
α-class	CA2; TaCA-His	0.47	0.74	0.61
α-class	CA3; PmaCA-His	0.49	0.81	0.65
CAs with high solubility scores				
α-class	CA6tr; SazCAtrunc-His	0.48	0.94	0.71
α-class	CA5; SspCA-His	0.53	0.94	0.74
γ-class	CA7; PabCA-His	0.70	0.92	0.81
γ-class	CA8; PhoCA-His	0.72	0.90	0.81

### Prediction of their solubility upon overexpression in *E. coli*

As described in Materials and methods, Protein-Sol and SOLpro programs were used to predict the solubility analysis of CAs upon overexpression in *E. coli*. The signal peptide-trimmed, His-tag-attached amino acid sequences of individual CAs (as listed in Table 1) were entered into the web servers. Their prediction score results were listed in Table 2. Specifically, the Protein-Sol program predicted solubility for the CA constructs was either close to (e.g., for the scaled solubility value, QuerySol for CA4 = 0.44) or greater than (QuerySol for CA1 to CA3, and CA5 to CA8 = 0.47 to 0.72) the population average solubility value (PopAvrSol) of 0.45 for the literature’s experimental solubility dataset [27]. Thus, the Protein-Sol program confirmed the soluble nature of the proposed expression for all CAs studied here, except CA4 (see Table 2, column 2).

Similarly, the SOLpro program predicted the solubility for the CA1 to CA8 in the range of 0.72 to 0.94, indicating that all of the CAs studied here would be soluble upon overexpression (Table 2). Note that for the SOLpro program, proteins with scores above 0.5 were evaluated as soluble upon overexpression [28].

Combining the predictions derived from these two programs, the average solubility scores were calculated for each of the CAs, and listed in the last column of Table 2. The CAs studied here are arranged in the order of average solubility scores from small to large. The results indicate that the CAs can be divided into two categories with relatively low average solubility scores (0.58 to 0.65 for CA4, CA1 to CA3) versus high total solubility scores (0.71 to 0.81 for CA5 to CA8).

### Designs ensuring the equal footing for the expression and comparison of different CAs

This study expressed all CAs in equal footing, with all signal peptides removed and with His-tag being added to C-terminal of all CAs. One extra benefit for adding His-tag to C-terminal is to ensure that all nickel column-purified proteins have full length catalytic domains with no degradation or incomplete translation.

In literature, TaCA was found to be one of the most thermostable CAs [4], but poorly expressed in *E. coli* [20]. Thus, in this study, TaCA was also expressed with N-terminal His-tag as a comparison pair, i.e. CA1-TaCA with N-terminal His-tag and CA2-TaCA with C-terminal His-tag (Table 1).

### Optimizing CA expression with Zn^2+^ supplement

The pET-28b-serial plasmids carrying CA genes (listed in Table 1) were transformed into BL21-CodonPlus (DE3)-RIL *E. coli* competent cells, and positive single colonies were picked from selection plates containing kanamycin (50 µg/mL) and chloramphenicol (30 µg/mL). CA-expressing *E. coli* transformants were cultured, and the cells were harvested for cell lysis, protein purification, downstream CA activity measurements, and thermostability characterization.

Zinc is one of the most widely occurring metal cofactors in enzymes and it is present in all CA families [29]. We speculated that the addition of ZnSO_4_ in the presence of IPTG would facilitate the normal folding and functionality of the expressed CAs. A final concentration of 0.5 mM ZnSO_4_ was chosen based on the literature [30] and we tested induction with 0.5 mM or 1.0 mM IPTG. It was found that 0.5 mM IPTG led to maximal expression levels of SspCA and SazCA, with no further enhancement of CA protein expression by using 1.0 mM IPTG. Thus, 0.5 mM ZnSO_4_ and 0.5 mM IPTG was used as an optimal condition for all CA expressions.

**Fig. 2:**
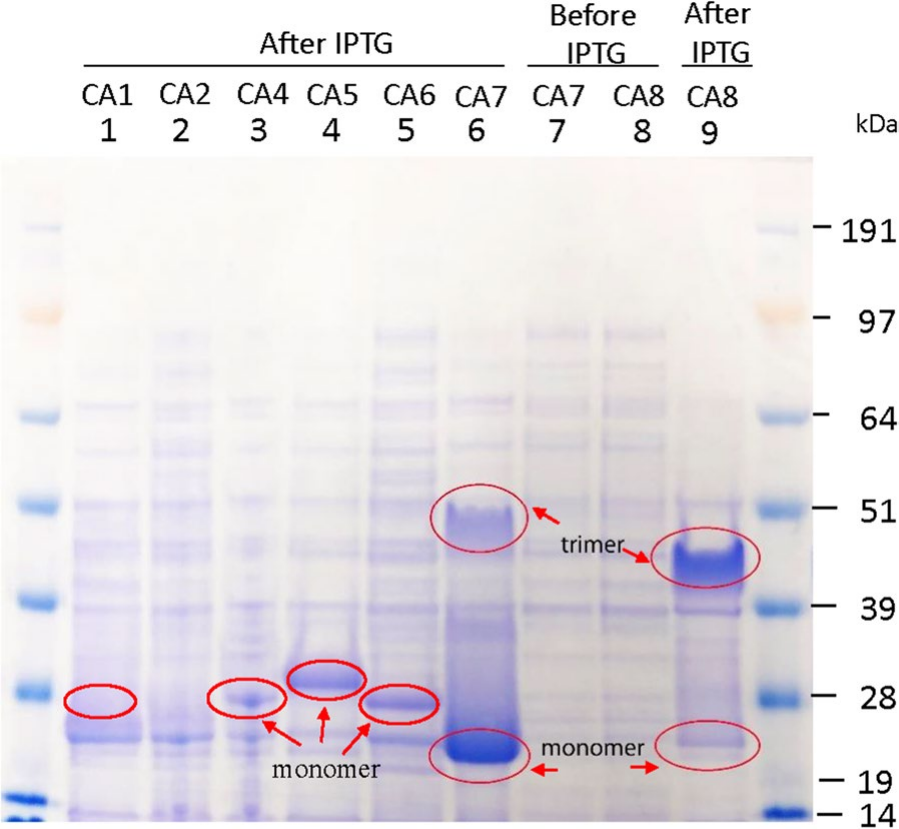
SDS-PAGE analysis of the cell lysates from the overexpression of CA1, CA2, and CA4 to CA8 in *E. coli*. CA1-TaCA with N-terminal his-tag had lower expression level than CA4 to CA8 (based on the protein bands that indicated by the red circles in individual lanes). CA1’s expression was confirmed by purification using Ni–NTA and SEC (see Supplementary Figure S1). The SDS-PAGE analysis of the cell lysate from the overexpression of CA3 was show in lane 1 of Supplementary Figure S2C

Comparing the SDS-PAGE results of cell lysates, it was noticed that when CA7 (i.e. PabCA) was expressed without additional Zn, it migrated as a major band on the gel corresponding to the MW of monomeric CA7, along with a lower intensity band corresponding to the MW of the presumed trimeric CA7 (Fig. 2**,** lane 6). Meanwhile, CA8 (i.e. PhoCA), another gamma-CA similar to PabCA, was expressed with the addition of 0.5 mM ZnSO_4_ and the major band on the SDS PAGE corresponded to the theoretic size of CA8 trimer (Fig. 2, lane 9). We believe that the presence of Zn leads to the formation of a more stable trimer for the gamma-CAs. Future studies are needed to investigate if disulfide bonds are formed between monomers for the putative trimer observed herein.

### Purification of five α-class CA proteins

Given the importance of the top thermostable and active α-class CAs, we provide a detailed description and discussions on their protein expression patterns in the following sections.

(1) CA1-TaCA

CA1-TaCA with an N-terminal His-tag exhibited a lower expression level compared to CA4 to CA8 (as indicated by the red circles on the protein bands in the individual lanes for these CAs in Fig. 2). The expression of CA1-TaCA was confirmed through purification using Ni–NTA and SEC (see Supplementary Figure S1). Note that since there was no significant difference in their SDS-PAGE profiles between CA1-TaCA and CA2-TaCA (lanes 1 and 2, respectively, in Fig. 2), CA2-TaCA with C-terminal His-tag was not pursued further.

(2) CA3-PmaCA as a highly thermostable CA

PmaCA, a highly thermostable α-carbonic CA, was discovered from a marine chemolithoautotrophic thermophile *Persephonella marina*; it was PBD: 6IM0_1 [25, 26]. It displayed a high thermostability up to 100 °C and broad pH tolerance (pH 4–10) [25, 26]**.**

In this study, CA3-PmaCA protein was purified via affinity chromatography using a Ni–NTA column, followed by SEC, as illustrated in Supplementary Figure S2. Multiple peaks on SEC chromatogram combined with low yield (1 mg L^−1^) prevented us from assaying this protein. The low yield of PmaCA in this study is consistent with the reported low yield of PmaCA protein expressed in *E. coli* strain BL21DE3 in literature, by which under the optimized protein expression conditions, Kanth et al*.* (2014) reported a yield of 1.29 L^−1^ mg, and 9.30 mg L^−1^ purified PmaCA and PmaCA (sp) from flask culture, respectively [26].

It is noteworthy that a more recent literature reported 15 mg per 100 mL for PmaCA expression with a C-terminal StrepTag in *E. coli* (equivalent to 150 mg L^−1^) [31]. Future studies are needed to investigate if different tags (i.e. C-terminal his-tag in this study versus C-terminal StrepTag in [31]) can lead to different expression yields of PmaCA in *E. coli*.

(3) CA4-LOGACA as a highly thermostable CA at alkaline pH

LOGACA was identified from a metagenome derived from an active hydrothermal vent chimney collected from the Logatchev hydrothermal field (PDB code 6EKI) [21]. The protein sequence of this CA had 83% identity to PmaCA [21], and had activity comparable to that of PmaCA [25]. It was previously expressed in *Bacillus subtilis* with a *Bacillus* signal peptide but without a His-tag, and purified using ion-exchange chromatography (SP-Sepharose) [21]. In this study, CA4-LOGACA protein was purified via affinity chromatography using a Ni–NTA column, and followed by SEC (Supplementary Figure S3). The purest sample (C8 peak from SEC; Supplementary Figure S3A) precipitated shortly after purification thus preventing us from assaying its activity. The low yield and multiple contaminants visible on SDS-PAGE could be a sign of expression/folding issues when this protein is overexpressed in *E. coli* (Supplementary Figure S3B).

(4) CA5-SspCA

As described in the Introduction, SspCA from *S. yellowstonense* YO3AOP1 can withstand temperatures up to 100 °C [6, 7]. This enzyme showed a good catalytic activity for the reaction of CO_2_ + H_2_O → HCO_3_^−^ + H^+^, with remarkable *k*_cat_ (9.35 × 10^5^ s^−1^) and *k*_cat_/*K*_M_ (1.1 × 10^8^ M^−1^ s^−1^) values, putting it among the most effective CAs known to date [6, 10].

We tried to purify CA5-SspCA with C-terminal 6xHis-tag by adjusting cell lysis and purification protocols. While we were able to obtain at least several milligrams of purified protein, each time the final SEC chromatogram looked different. Following are two representative but opposite examples of the SspCA SECs that selected from eight purification runs, which illustrate the variations in protein properties of SspCA (Supplementary Figure S4A-B). For the second example illustrated in Supplementary Figure S4B, the enzymatic activity of the smaller fraction “C3” was approximately 3 times of that for B10 peak although the major component of both fractions was CA; this observation was further discussed in detail in below section for the CA5 and CA6 enzyme activities.

It is noteworthy that the above observed changing SEC profiles of the protein elution could indicate underlying issues in protein folding and degradation, which could lead to different CA5 populations that have solubility issues (i.e. drag in SEC).

(5) CA6-SazCA as fastest, most effective CA

SazCA (i.e. CA6 in this study) from the extremophilic bacterium *S. azorense* was originally isolated from terrestrial hot springs in the Azores and is known to be the fastest wild-type carbonic anhydrase [8], and the second most efficient enzyme (after superoxide dismutase), with turnover number k_cat_ of 4.4 × 10^6^ s^−1^ [8, 10], and a *k*_cat_/*K*_M_ value of 3.5 × 10^8^ M^−1^ s^−1^ [10]. In addition, it is highly thermostable, and can endure 100 °C for a prolonged period (more than 3 h) and still maintain good catalytic activity [8, 10]. These features put CA6-SazCA on par with the CA5-SspCA among the most effective CAs known to date.

**Fig. 3:**
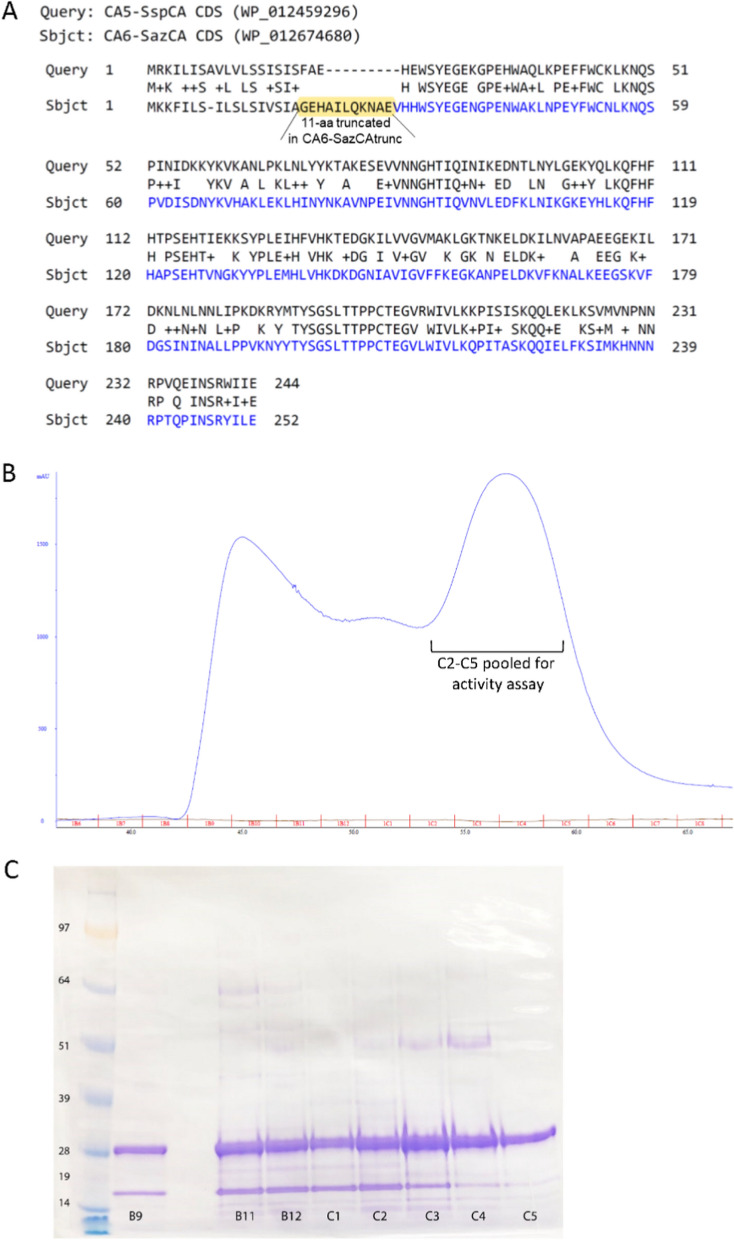
CA6-SazCAtruc protein sequence feature and purification. **A** Amino acid sequence alignment for the CDS of full length SspCA and SazCAfull, including their signal sequences. A 11-amino acid sequence (highlighted in yellow) was removed from SazCAfull to obtain CA6-SazCAtrunc (in blue text). **B** SEC chromatogram for CA6-SazCAtrunc. **C** SDS PAGE for fractions B9-C5 from CA6-SazCAtruc SEC purification

In this study, when we designed the CA6-SazCA construct, we not only removed the signal peptide, but also removed the 11 a.a. residues (GEHAILQKNAE) that followed the signal sequence based on an alignment with the SspCA (Fig. 3A). Our intention was to evaluate if this 11-amino acid fragment plays a role in the reported high enzymatic activity of this CA.

Protein was purified via affinity chromatography using Ni–NTA column, followed by SEC (Fig. 3B). Fractions B9-B11 collected from SEC precipitated shortly after purification. Fractions C2-C5 were pooled for activity measurements, as described in the section below.

(6) Enzyme activities of CA5-SspCA and CA6-SazCAtrunc

As described in above sections, CA5-SspCA was reported to be the most thermostable CA and CA6-SazCA as the fastest CA activity reported so far, thus our enzymatic activity analyses for the purified proteins were focused on these two candidates in this study.

For CA5-SspCA, its activity was determined for 2 different fractions from SEC column as 409 WAU/mg for the 1st peak (i.e. B10 peak, Supplementary Figure S4B) and 1121 WAU/mg for the 2nd peak (i.e. C3 peak, Supplementary Figure S4B). For CA6-SazCAtrunc, SEC fractions C2–C5 were pooled (Fig. 3) for activity measurements, which showed a low activity at 240 WAU/mg. Such activity measurement results contradict previously published data. SazCAfull was reported to be 3× more active than SspCA but our data shows CA6-SazCAtrunc to be less active (240 WAU/mg for the SazCA vs. 409 or 1121 WAU/mg for different fractions of SspCA). These results suggest that the deletion of 11 a.a. residues (GEHAILQKNAE, Fig. 3A) had negative impact on the enzyme activity of CA6-SazCAtrunc. It is noteworthy that future studies are needed to design and express CA6-SazCAfull using the same procedures employed in this study. This will enable a direct comparison of the enzymatic activities between CA6-SazCAfull and CA6-SazCAtrunc proteins.

### Aggregation issues in certain α-class CAs after purification

As described in the purification results section, precipitation was observed in the purified fractions of both CA4-LOGACA and CA6-SazCA shortly after purification. Further experiments, beyond the scope of this paper, are needed to fully understand this protein aggregation issue. Over the past few decades, many studies have explored the use of peptide- or protein-based tags to minimize aggregation and enhance solubility of heterologous proteins expressed in *E*. *coli* with minimal or no impact on enzyme activity [32]. Tag systems such as MBP (maltose binding protein) [33] and NEXT (originating from N-terminus of *Hydrogenovibrio marinus* CA) [34] are widely used. NEXT tag was shown to increase the soluble expression level in E. coli BL21 (DE3) by up to 5.6–8.3 fold [32]. It is possible that CA4-LOGACA and CA6-SazCA would show improved activity and/or protein titer with properly optimized fusion tag strategies.

### CA7-PabCA and CA8-*PhoCA* in γ-class

As described in an earlier section and in Table 1, CA7-PabCA was a γ-class CA from hyperthermophilic archaea *Py. abyssi* (GenBank accession no. WP_010867695), while CA8-PhoCA was another γ-class CA from hyperthermophilic archaea *Py. horikoshii* (WP_010885665). These two γ-class CAs have 92% identities in their amino acid sequences. First, we applied the cell lysate to the Ni–NTA column and for both PabCA and PhoCA while large quantities of protein eluted, the protein fractions were of bright blue color, believed to be Ni metal stripped from the Ni–NTA column. We tried to remove Ni from the PabCA and PhoCA samples by incubating first with 50 mM EDTA and running the samples through desalting column—unsuccessfully. Bright blue color persisted in the samples. Raising the EDTA concentration to 100 mM led to precipitation of both proteins. Since we believe that presence of a foreign metal ion like Ni in the active site could significantly affect the activity, we decided to change the purification protocol.

Next, we applied the cell lysate to a Cobalt His-pur column, another His-tag affinity column that utilizes cobalt instead of nickel. After elution from Co His-pur column, protein fractions were concentrated and applied to HiLoad Superdex 75 size exclusion column, and the SEC chromatography for CA7-PabCA and CA8-PhoCA were shown in Supplementary Figure S5A and B, respectively.

γ-CAs were reported to be active as homotrimers [25], and indeed our SDS-PAGE analysis of both the cell lysates (Fig. 1, lanes 6 and 9) and the SEC fractions (Supplementary Figure S5C) showed the presence of protein bands with the expected size of trimers for CA7 and CA8. It is noteworthy that most α-CAs are primarily monomers [35, 36], with a few reported cases of dimeric α-CAs, such as CA5-SspCA [37], and hCAs IX and XII. Both hCAs IX and XII can also function as monomers [36]. In this study, α-CAs (i.e., CA1, CA3 to CA6) were found to be in monomer form based on size exclusion analysis of purified CAs (Supplementary Figures S1 to S4). The quaternary structures of α- and γ-CAs are quite different. Given the significant differences in the expression titers observed between α- and γ-CAs in this study, future research is needed to investigate if there is a connection between their quaternary structures and solubility. There are conflicting reports for the activities of PhoCA in literature. Previously, the purified PhoCA with *E. coli* as expression host was found to have no activity [18]. However, recently, the same wild-type PhoCA protein expressed in *E. coli* with C-terminal His-tag and purified by binding to Ni-column, and was found to have esterase activity at 37 °C, 45 °C and 70 °C [19].

In this study, the purified PabCA and PhoCA protein did not show any enzymatic activity. The probable reasons for this might be due to the potential incorporation of foreign metals like Ni or Co during the affinity purification steps. In addition, it was reported that both Zn^2+^- and Ca^2+^-binding sites in the crystal structure of PhoCA are needed for activity [18]. Thus, the assay conditions may also need to be optimized for the estimation of γ-CA proteins.

### Summary of CA purification titers and specific activities

The CA expression titers and specific activities are summarized in Table 3 for each CA protein. Note that the two γ-CAs had the highest purification titers (20 and 75 mg L^−1^ CA7 and CA8, respectively), Since high purification levels are important for downstream scale-up protein production and application, future studies are needed to solve the issue for functionality analysis of the expressed γ-CAs, as these two CAs have great potential due to their high expressibility.

**Table 3:** Comparison of CA expression titers and specific activities

Enzymes	Titers (mg L^−1^)	Specific activities
CA3-PmaCA	1	N/A due to low protein yield
CA4-LOGACA	2	N/A due to protein precipitation
CA5-SspCA	5	409 WAU/mg for SEC fraction B10; 1121 WAU/mg for SEC fraction C3
CA6-SazCAtrunc	10	240 WAU/mg for pooled SEC fraction C2-C5
CA7-PabCA	20	Undetected
CA8-PhoCA	75	Undetected

Expression titers of the six CAs were determined based on purified enzyme amounts, in line with literature [31].

### Success rates for the solubility prediction for CA expression

In this study, six constructs (CA3 to CA8) out of eight CA constructs were experimentally proven to be soluble, which led to a successful prediction rate of 75% for SOLpro. This prediction accuracy rate is very close to the reported accuracy rate of 74% while the SOLpro was originally published [16]. Meanwhile, Protein-Sol program also correctly predicted that CA3, CA5 to CA8 were soluble upon overexpression, and more impressively, it assigned the highest solubility scores of 0.70 and 0.72 to CA7 and CA8, respectively, which turned out to be the two CAs with the highest expression titers (as discussed below in the phylogenetic tree section).

Together, these two programs had good correct prediction rates for the set of CAs in this study, and using a combination of these two different prediction programs should increase the overall prediction accuracy.

Nevertheless, our CA protein dataset can be used for the further retraining of machine learning methods to predict the solubility of overexpressing proteins, as enlarging sample size will benefit machine learning studies.

### Phylogenetic tree analysis and its correlation with the expression of selected CAs

**Fig. 4:**
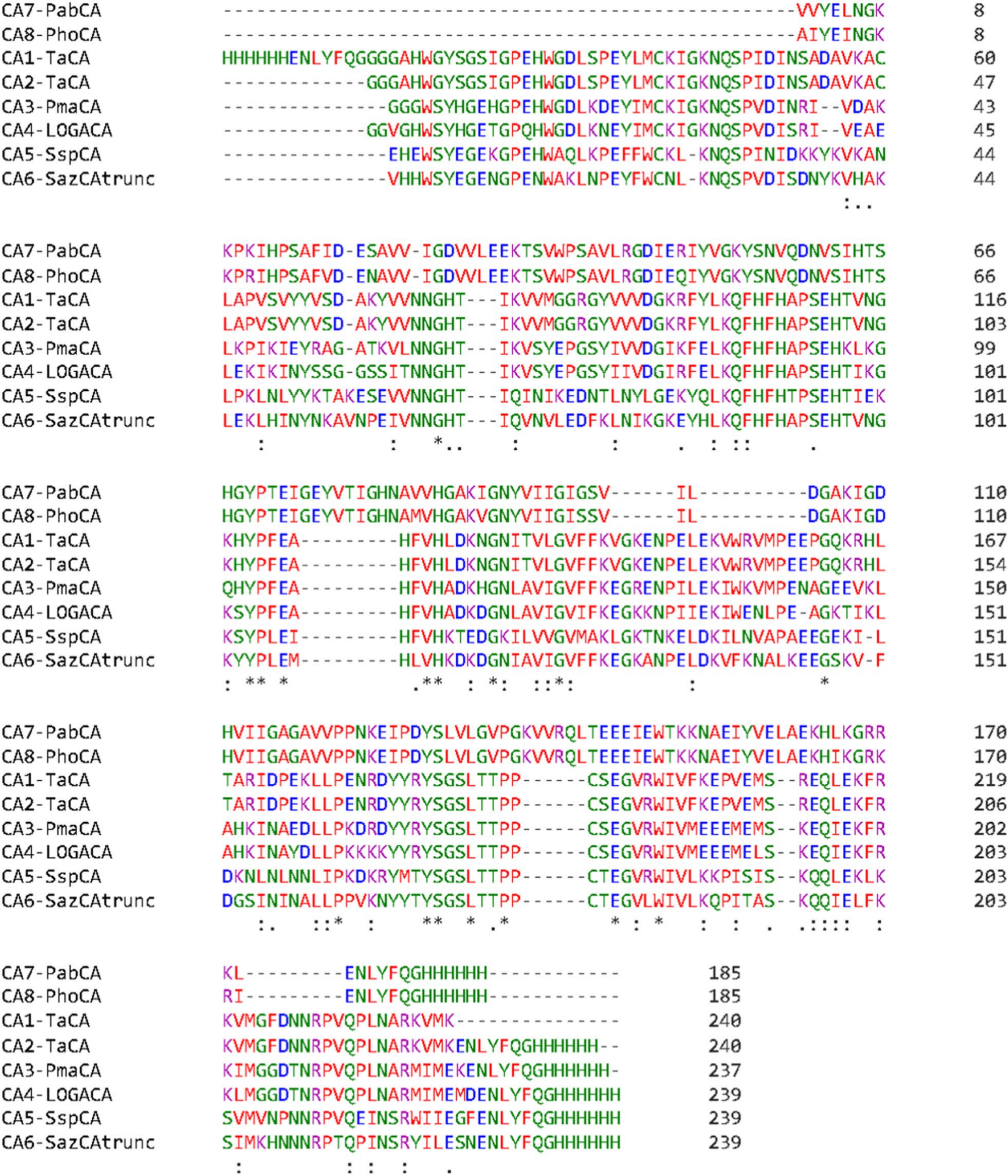
Multiple sequence alignment of CA1 to CA8 using Clustal Omega

Phylogenetic analyses of protein expression are useful to address a range of questions. For example, such analyses can be useful to identify proteins with evolutionary shifts in expression that correlate with evolutionary changes in physiological and functional characters of interest. In this study, the multiple sequence alignment and phylogenetic tree analysis of CAs were conducted by using Clustal Omega [38]. The multiple sequence alignment of CAs is illustrated in Fig. 4.

**Fig. 5:**
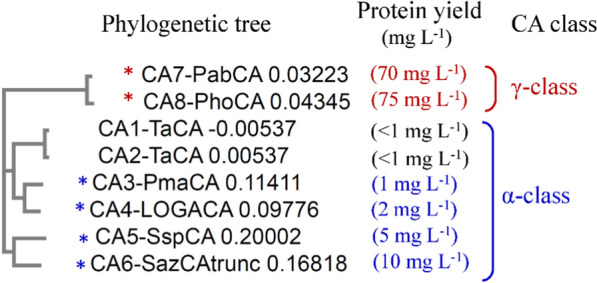
Phylogenetic analysis of CAs. The amino acid sequences were aligned, and the phylogenetic tree was built by using Clustal Omega. Neighbour-joining tree without distance corrections was selected. CA1-TaCA and CA2-TaCA represent the TaCA expressed with N- and C-terminal His-tag, respectively

The phylogenetic tree generated by Clustal Omega (Neighbour-joining without distance corrections) is shown in Fig. 5. It shows that for the α-CAs, CA3 and CA4 are closely related and CA5 and CA6 are closely related. In contrast, CA1 and CA2 are only distantly related to CA3 and CA4 (Fig. 8). For the γ-CAs, CA7 and CA8 are also closely related with each other, and distinct from all other α-CAs.

Our analysis also demonstrated that there is a correlation between phylogenetic tree and the expression titers of CAs. Specifically, the gamma class of CAs (CA7 and CA8, 70 and 75 mg L^−1^, respectively) had significantly higher expression titers than that of alpha class of CAs (CA3 to CA6, 1 to 10 mg L^−1^). Furthermore, the four CAs with highest titers (CA5 to CA8, 5 mg/L to 75 mg/L, Fig. 7) had also the highest values of average solubility scores in the range of 0.71 to 0.81 (Table 2, last column). Such clustering of protein expression patterns indicates that the folding and structure of CAs is class-dependent.

### Alternative experimental procedures

It is noteworthy that several previous studies did not employ Western blot analysis to screen for heterologous CA protein expression. Instead, those studies directly subjected cell lysates or crude protein preparations to Ni–NTA for His-tagged CAs or to Strep-tag columns for Strep-tagged CAs, followed by IEC and/or SEC chromatography and enzyme activity analyses [20, 26, 31]. This study followed similar procedures: seven CA proteins were expressed and then subjected to Ni–NTA and SEC chromatography, with SDS-PAGE analyses. The corresponding data are presented in Supplemental Figs. S1 to S5 and Fig. 3.

Nevertheless, future studies could explore peptide- or protein-fusion techniques to enhance protein solubility and production yield. Western blot analysis remains a viable method for quickly screening heterologous CA expression. If SDS-PAGE and Western blot analyses do not indicate enhanced expression levels, there would be no need to undertake the time- and labor-intensive steps of affinity column purification followed by IEC or SEC.

## Conclusions

The prediction and experimental validation of protein solubility are important tools for improving the production of soluble and active recombinant proteins in *E. coli*. Overall, the expression of CA constructs in *E. coli* was partially successful. We were able to express CA5-SspCA, CA6-SazCAtrunc, CA7-PabCA and CA8-PhoCA with yields of 5, 10, 20 and 75 mgs of purified protein from 1L of culture, respectively. The expression titers were in close correlation with their bioinformatically predicted protein solubility scores. In addition, a CA class-specific clustering pattern for protein solubility and production yields was observed based on phylogenetic tree analysis.

Enzymatic activity analyses of purified enzymes in this study showed that **(1)** as the known most thermostable CA, CA5-SspCA had two distinctive SEC peaks with 3 times difference in activity; and that **(2)** as the known fastest, most effective CA, the 11-a.a. after the signal sequence in CA6-SazCA may be essential for its activity. Therefore, future studies are warranted to express CA6-SazCAfull, allowing a direct comparison of the enzymatic activities between CA6-SazCAfull and CA6-SazCAtrunc proteins.

In addition, more work is needed to **(1)** conduct the N-terminal amino acid sequencing of the purified proteins to confirm the integrity of the full-length translated CA-7 and CA-8 proteins, even though the likelihood of unwanted N-terminal cleavage is rare, and **(2)** optimize assay conditions for measuring the enzyme activity of CA8-PhoCA, ensuring the appropriate concentrations for both Zn^2+^ and Ca^2+^ ions required for its activity [18]. Equally important, the approaches explored and the knowledge gained in this study should be applicable to other target proteins and other host bacterial species.

### Supplementary Information

Supplementary material 1.

## Data Availability

The data supporting the results of this article are included in this published article [and its supplementary information file].

## Abbreviations

CA: Carbonic anhydrase
CA1-TaCA: CA from *Themovibrio ammonificans* with N-terminal His-tag
CA2-TaCA: CA from *Themovibrio ammonificans* with C-terminal His-tag
CA3-PmaCA: CA from *Persephonella marina*
CA4-LOGACA: CA from Logatchev hydrothermal field
CA5-SspCA: CA from *Sulfurihydrogenibium *sp*. (i.e. Sulfurihydrogenibium yellowstonense* YO3AOP1
CA6-SazCAtrunc: CA from *Sulfurihydrogenibium azorense*, truncated form
CA7-PabCA: CA from *Pyrococcus abyssi*
CA8-PhoCA: CA from *Pyrococcus horikoshii*
LOGACA: Logatchev hydrothermal field carbonic anhydrase
MW: Molecular weight
