# Supplementary material for: Streamlining heterologous expression of top carbonic anhydrases in Escherichia coli: bioinformatic and experimental approaches
Source: Microb Cell Fact. 2024 Jul 2;23:190. doi: 10.1186/s12934-024-02463-5 (PMC11218372; doi:10.1186/s12934-024-02463-5)
Supplement: Supplementary file 1 — Supplementary material 1. [file 12934_2024_2463_MOESM1_ESM.docx]

**Additional file 1**

File type: PDF

**Title: Streamlining the heterologous expression of top carbonic anhydrases in *Escherichia coli*:**

**Bioinformatic and experimental approaches**

## Hui Wei^1,§,*^, Vladimir V. Lunin^1,§^, Markus Alahuhta^1^, Michael E. Himmel^1^, Shu Huang^1,†^, Yannick J. Bomble^1^, Min Zhang^1,*^

**^1^**Biosciences Center, National Renewable Energy Laboratory, Golden, CO 80401, USA

**^2^**National Bioenergy Center, National Renewable Energy Laboratory, Golden, CO 80401, USA

**List of contents:**

1. Supplementary Figure S1. CA1-TaCA purification.

2. Supplementary Figure S2. CA3-PmaCA purification.

3. Supplementary Figure S3. CA4-LOGACA purification.

4. Supplementary Figure S4. CA5-SspCA purification.

5. Supplementary Figure S5. CA7-PabCA and CA8-PhoCA purification.

6. Sequences of CAs expressed in *E. coli.*

**
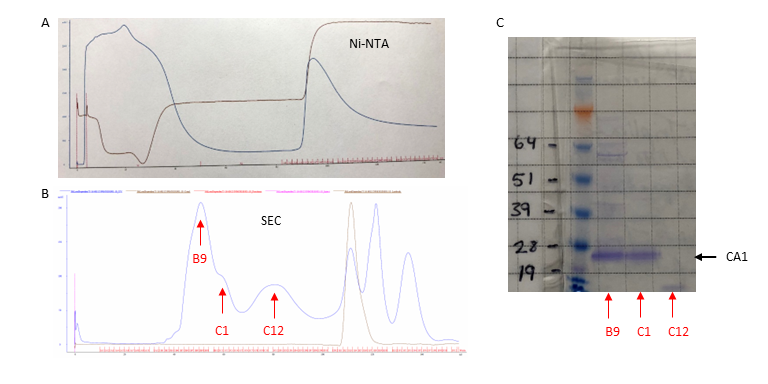
**

**Supplementary Figure S1**. **Purification of CA1-TaCA with N-terminal his-tag**. **(A) Ni-NTA** chromatogram. **(B)** SEC chromatogram. **(C)** Fractions from the SEC were checked on the SDS-PAGE.


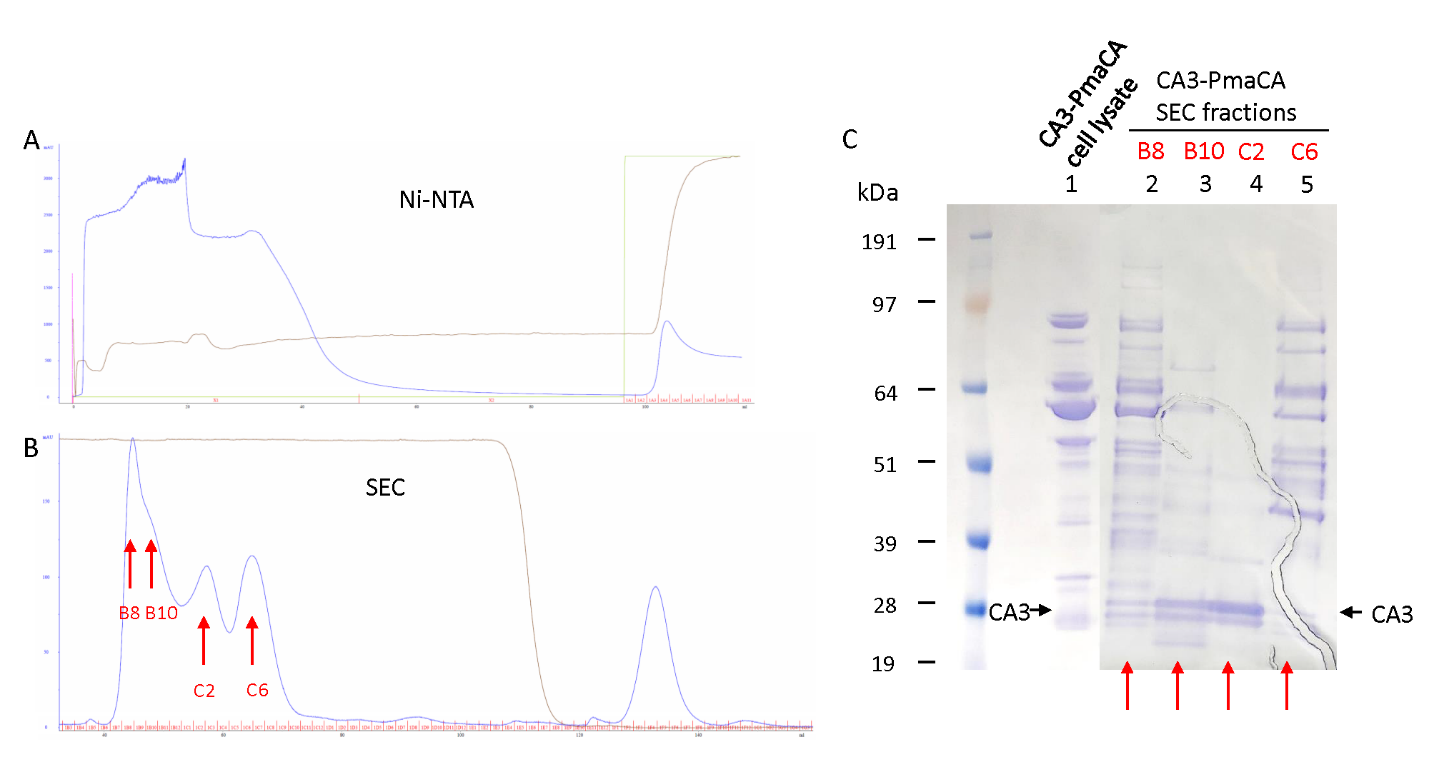


**Supplementary Figure S2**. **CA3-PmaCA purification**. **(A)** Ni-NTA chromatogram. **(B)** SEC chromatogram. **(C)** Fractions from the SEC for CA3-PmaCA purification were checked on the SDS-PAGE; lane 1 shows the SDS-PAGE analysis of the cell lysates from the overexpression of CA3.


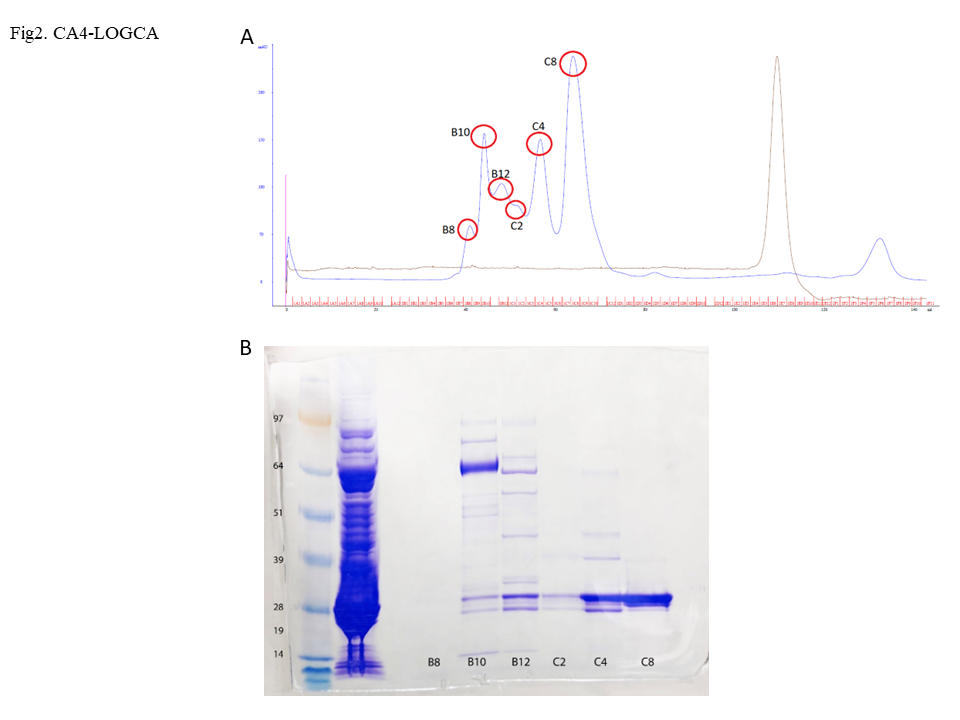


**Supplementary Figure S3. CA4-LOGACA purification. (A)** SEC chromatogram for CA4-LOGACA purification. **(B)** Fractions from the SEC for CA4-LOGACA were checked on the SDS-PAGE.


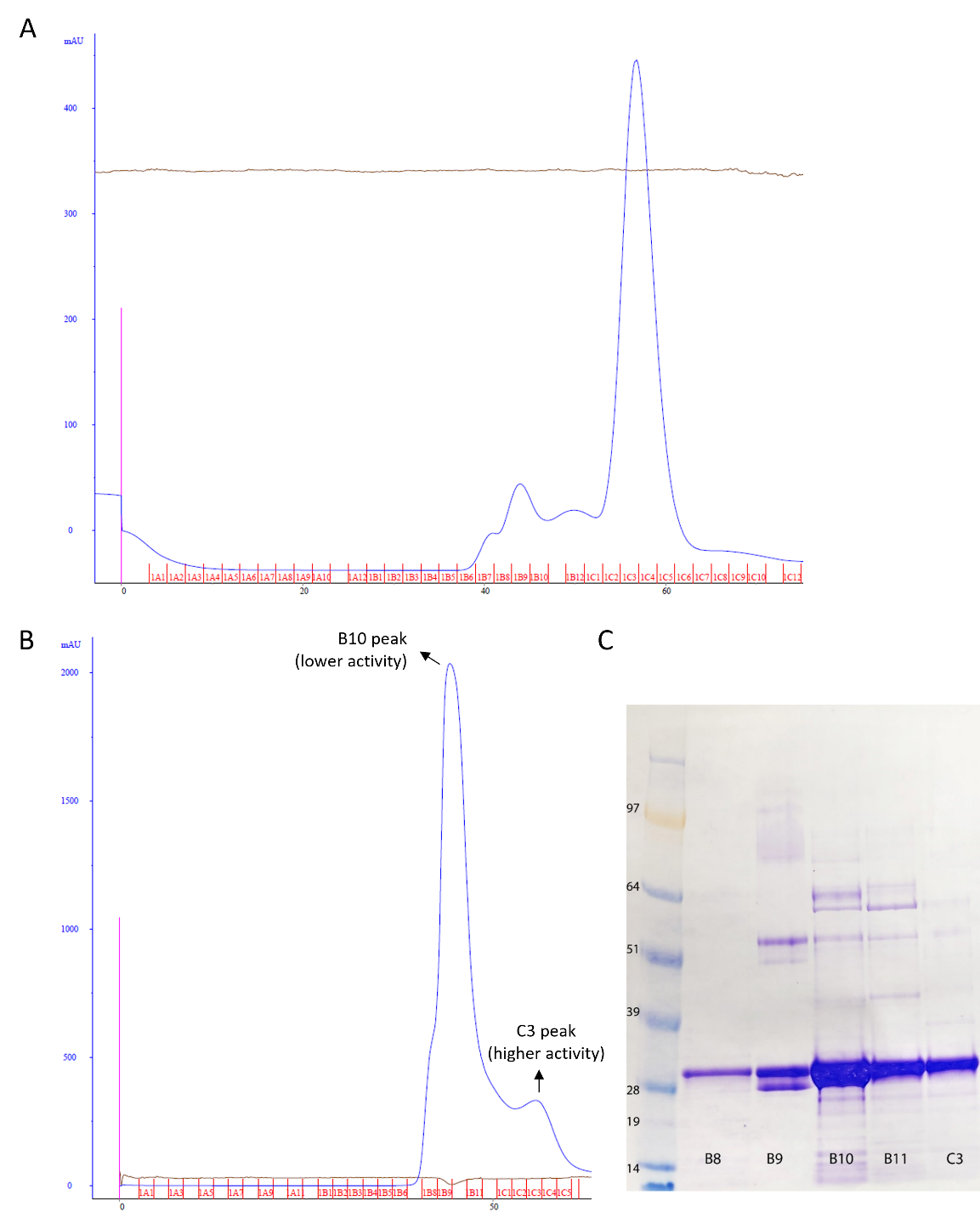


**Supplementary Figure S4. CA5-SspCA purification.** **(A-B)** SEC runs for two different CA5-SspCA purification attempts. **(C)** SDS-PAGE for the fractions B8-B11 and C3 from the final SEC run of CA5-SspCA purification.


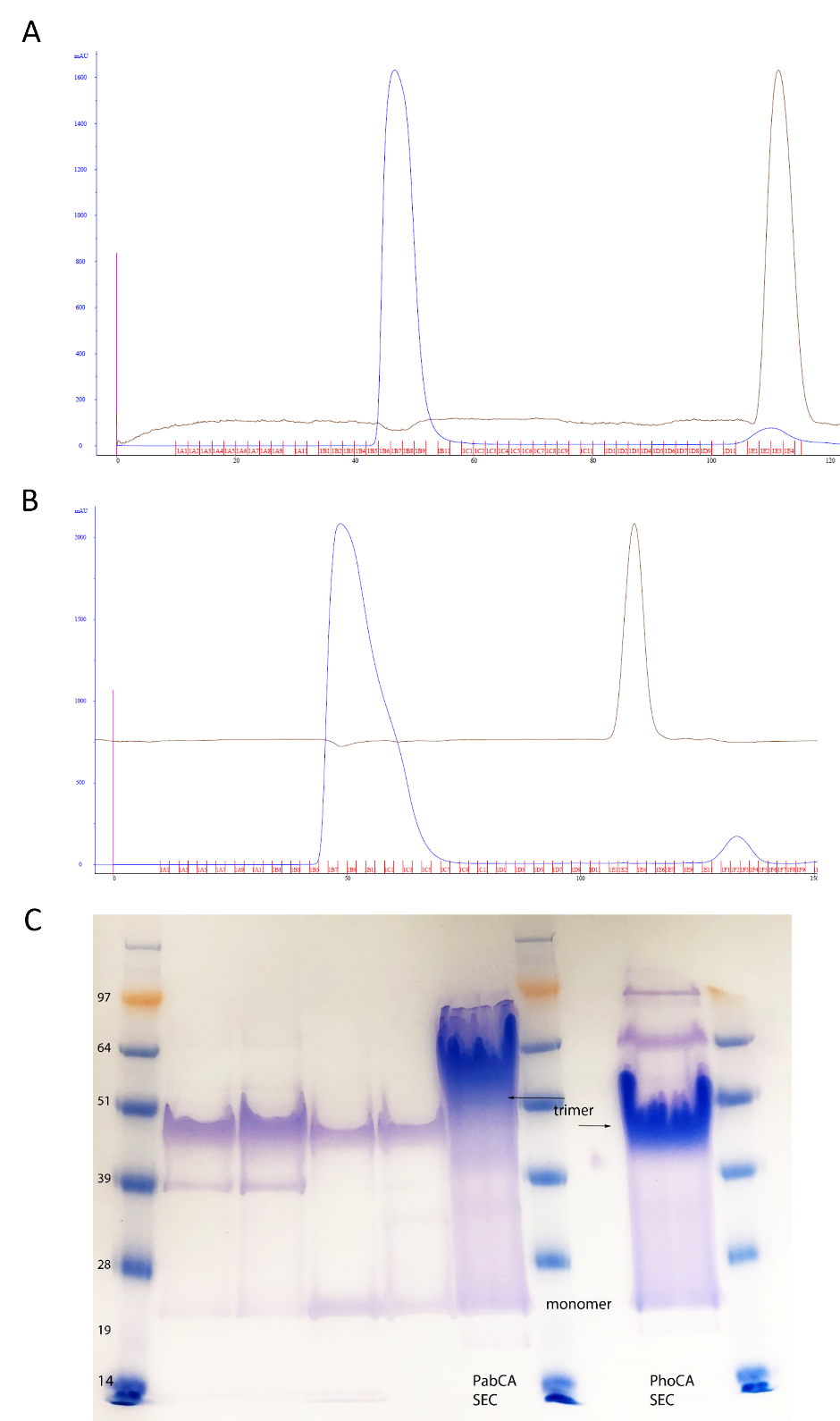


**Supplementary Figure S5.** **CA7-PabCA and CA8-PhoCA purification.** **(A)** PabCA SEC purification. **(B)** PhoCA SEC purification**. (C)** SDS-PAGE for PabCA and PhoCA.

**Sequences of CAs expressed in *E. coli***

**Note:** The DNA sequence of synthesized genes were codon-optimized using *E. coli* codon usage frequency.

As described in the Materials and Methods section, eight CA-expressing constructs were designed and built, as described below:

**SEQ ID: CA1 for *E. coli***

**SEQ name: HisTag-TEV-TaCA**

TYPE: PRT; DNA

**PRT sequence** (240 aa; pI, 8.96; MW, 27.5 kDa):

>CA1-**TaCA**

HHHHHHENLYFQGGGGAHWGYSGSIGPEHWGDLSPEYLMCKIGKNQSPIDINSADAVKACLAPVSVYYVSDAKYVVNNGHTIKVVMGGRGYVVVDGKRFYLKQFHFHAPSEHTVNGKHYPFEAHFVHLDKNGNITVLGVFFKVGKENPELEKVWRVMPEEPGQKRHLTARIDPEKLLPENRDYYRYSGSLTTPPCSEGVRWIVFKEPVEMSREQLEKFRKVMGFDNNRPVQPLNARKVMK

**DNA sequence** (737 bp; used to generate plasmid Gene1-pET-28a-HisTag-TEV-TaCA):

Start with: ccatgggg,

CATCATCATCATCATCACGAGAACCTGTATTTTCAGGGTGGCGGTGGCGCGCACTGGGGTTATAGCGGTAGCATTGGTCCGGAGCACTGGGGTGACCTGAGCCCGGAATACCTGATGTGCAAGATTGGCAAAAACCAAAGCCCGATCGACATTAACAGCGCGGATGCGGTTAAGGCGTGCCTGGCGCCGGTTAGCGTGTACTATGTGAGCGACGCGAAGTATGTGGTTAACAACGGTCACACCATCAAAGTGGTTATGGGTGGCCGTGGCTACGTGGTTGTGGATGGCAAGCGTTTTTATCTGAAACAGTTCCACTTTCACGCGCCGAGCGAGCACACCGTTAACGGCAAGCACTACCCGTTCGAAGCGCACTTTGTGCACCTGGACAAAAACGGCAACATTACCGTTCTGGGTGTGTTCTTTAAGGTTGGCAAAGAGAACCCGGAGCTGGAAAAGGTTTGGCGTGTGATGCCGGAGGAACCGGGTCAAAAGCGTCACCTGACCGCGCGTATCGACCCGGAGAAACTGCTGCCGGAAAACCGTGATTACTATCGTTATAGCGGTAGCCTGACCACCCCGCCGTGCAGCGAAGGCGTTCGTTGGATTGTGTTCAAAGAGCCGGTTGAAATGAGCCGTGAGCAGCTGGAAAAGTTCCGTAAAGTGATGGGTTTTGATAACAACCGTCCGGTTCAACCGCTGAACGCGCGTAAGGTGATGAAA,

End with: taactcgag

**SEQ ID: CA2 for *E. coli***

**SEQ name: TaCA-TEV**

TYPE: PRT; DNA

**PRT sequence** (240 aa; pI, 8.96; MW, 27.5 kDa):

>CA2-TaCA

GGGAHWGYSGSIGPEHWGDLSPEYLMCKIGKNQSPIDINSADAVKACLAPVSVYYVSDAKYVVNNGHTIKVVMGGRGYVVVDGKRFYLKQFHFHAPSEHTVNGKHYPFEAHFVHLDKNGNITVLGVFFKVGKENPELEKVWRVMPEEPGQKRHLTARIDPEKLLPENRDYYRYSGSLTTPPCSEGVRWIVFKEPVEMSREQLEKFRKVMGFDNNRPVQPLNARKVMKENLYFQGHHHHHH

**DNA sequence** (716 bp; used to generate plasmid Gene2-pET-28b-TaCA-TEV-HisTag):

Start with: ccatgggc

GGTGGCGGTGCGCACTGGGGCTACAGCGGTAGCATTGGTCCGGAGCACTGGGGCGACCTGAGCCCGGAATATCTGATGTGCAAGATTGGTAAAAACCAGAGCCCGATCGACATTAACAGCGCGGATGCGGTTAAGGCGTGCCTGGCGCCGGTTAGCGTGTACTATGTGAGCGACGCGAAGTACGTGGTTAACAACGGCCACACCATCAAAGTGGTTATGGGCGGTCGTGGTTACGTGGTTGTGGATGGCAAGCGTTTCTATCTGAAACAATTCCACTTTCACGCGCCGAGCGAGCACACCGTTAACGGCAAGCACTATCCGTTCGAAGCGCACTTTGTGCACCTGGACAAAAACGGTAACATTACCGTTCTGGGCGTGTTCTTTAAGGTTGGTAAAGAGAACCCGGAGCTGGAAAAGGTTTGGCGTGTGATGCCGGAGGAACCGGGTCAGAAGCGTCACCTGACCGCGCGTATCGACCCGGAGAAACTGCTGCCGGAAAACCGTGATTACTATCGTTATAGCGGTAGCCTGACCACCCCGCCGTGCAGCGAGGGTGTTCGTTGGATTGTGTTCAAAGAGCCGGTTGAAATGAGCCGTGAGCAACTGGAAAAGTTCCGTAAAGTGATGGGCTTTGATAACAACCGTCCGGTTCAGCCGCTGAACGCGCGTAAGGTGATGAAAGAAAACCTGTATTTTCAAGGT,

End with: ctcgag

**SEQ ID: CA3 for *E. coli***

**SEQ name: PmaCA-TEV**

TYPE: PRT; DNA

**PRT sequence** (237 aa; pI, 6.86; MW, 27.4 kDa):

>CA3-PmaCA

GGGWSYHGEHGPEHWGDLKDEYIMCKIGKNQSPVDINRIVDAKLKPIKIEYRAGATKVLNNGHTIKVSYEPGSYIVVDGIKFELKQFHFHAPSEHKLKGQHYPFEAHFVHADKHGNLAVIGVFFKEGRENPILEKIWKVMPENAGEEVKLAHKINAEDLLPKDRDYYRYSGSLTTPPCSEGVRWIVMEEEMEMSKEQIEKFRKIMGGDTNRPVQPLNARMIMEKENLYFQGHHHHHH

**DNA sequence** (707 bp; used to generate plasmid Gene3-pET-28b-PmaCA-TEV-HisTag):

Start with: ccatgggc

GGTGGCGGTTGGAGCTACCACGGCGAACATGGTCCGGAGCACTGGGGTGACCTGAAAGATGAGTATATCATGTGCAAGATTGGCAAAAACCAGAGCCCGGTGGACATCAACCGTATTGTTGATGCGAAGCTGAAACCGATCAAGATTGAGTACCGTGCGGGTGCGACCAAAGTGCTGAACAACGGCCACACCATCAAAGTTAGCTACGAACCGGGTAGCTATATCGTGGTTGACGGCATTAAGTTCGAACTGAAACAGTTCCACTTTCACGCGCCGAGCGAGCACAAGCTGAAAGGTCAACACTATCCGTTCGAAGCGCACTTTGTGCACGCGGATAAACACGGCAACCTGGCGGTGATTGGTGTTTTCTTTAAGGAAGGCCGTGAGAACCCGATCCTGGAAAAGATTTGGAAAGTGATGCCGGAGAACGCGGGCGAGGAAGTTAAGCTGGCGCACAAAATCAACGCGGAAGACCTGCTGCCGAAGGACCGTGATTACTATCGTTACAGCGGTAGCCTGACCACCCCGCCGTGCAGCGAGGGCGTGCGTTGGATTGTTATGGAGGAAGAGATGGAAATGAGCAAAGAACAAATCGAGAAGTTCCGTAAAATTATGGGCGGTGATACCAACCGTCCGGTTCAGCCGCTGAACGCGCGTATGATCATGGAAAAGGAGAACCTGTATTTTCAAGGT,

End with : ctcgag

**SEQ ID: CA4 for *E. coli***

**SEQ name: LOGACA-TEV**

TYPE: PRT; DNA

**PRT sequence** (239 aa; pI, 7.93; MW, 27.3 kDa):

>CA4-**LOGACA**

GGVGHWSYHGETGPQHWGDLKNEYIMCKIGKNQSPVDISRIVEAELEKIKINYSSGGSSITNNGHTIKVSYEPGSYIIVDGIRFELKQFHFHAPSEHTIKGKSYPFEAHFVHADKDGNLAVIGVIFKEGKKNPIIEKIWENLPEAGKTIKLAHKINAYDLLPKKKKYYRYSGSLTTPPCSEGVRWIVMEEEMELSKEQIEKFRKLMGGDTNRPVQPLNARMIMEMDENLYFQGHHHHHH

**DNA sequence** (713 bp; used to generate plasmid Gene4-pET-28b-LOGACA-TEV-HisTag):

Start with : ccatgggc

GGTGGCGTGGGTCACTGGAGCTACCACGGTGAAACCGGTCCGCAACACTGGGGCGACCTGAAGAACGAATATATCATGTGCAAGATTGGTAAAAACCAGAGCCCGGTGGATATCAGCCGTATTGTTGAAGCGGAGCTGGAAAAGATCAAAATTAACTACAGCAGCGGTGGCAGCAGCATCACCAACAACGGCCACACCATTAAAGTGAGCTACGAGCCGGGCAGCTATATCATTGTTGACGGTATCCGTTTCGAACTGAAACAATTCCACTTTCACGCGCCGAGCGAGCACACCATTAAGGGTAAAAGCTATCCGTTCGAAGCGCACTTTGTGCACGCGGACAAGGATGGTAACCTGGCGGTGATCGGCGTTATTTTTAAGGAAGGTAAGAAAAACCCGATCATTGAGAAAATCTGGGAAAACCTGCCGGAGGCGGGCAAGACCATCAAACTGGCGCACAAAATTAACGCGTACGATCTGCTGCCGAAGAAAAAGAAATACTATCGTTATAGCGGTAGCCTGACCACCCCGCCGTGCAGCGAGGGTGTGCGTTGGATCGTTATGGAGGAAGAGATGGAACTGAGCAAAGAGCAGATTGAAAAGTTCCGTAAACTGATGGGTGGCGACACCAACCGTCCGGTTCAACCGCTGAACGCGCGTATGATCATGGAGATGGATGAAAACCTGTACTTTCAGGGT,

End with: ctcgag

**SEQ ID: CA5 for *E. coli***

**SEQ name: SspCA-TEV**

TYPE: PRT; DNA

**PRT sequence** (239 aa; pI, 9.04; MW, 27.8 kDa):

>CA5-**SspCA**

EHEWSYEGEKGPEHWAQLKPEFFWCKLKNQSPINIDKKYKVKANLPKLNLYYKTAKESEVVNNGHTIQINIKEDNTLNYLGEKYQLKQFHFHTPSEHTIEKKSYPLEIHFVHKTEDGKILVVGVMAKLGKTNKELDKILNVAPAEEGEKILDKNLNLNNLIPKDKRYMTYSGSLTTPPCTEGVRWIVLKKPISISKQQLEKLKSVMVNPNNRPVQEINSRWIIEGFENLYFQGHHHHHH

**DNA sequence** (713 bp; used to generate plasmid Gene5-pET-28b-SspCA-TEV-HisTag):

Start with: ccatgggc

GAGCACGAATGGAGCTACGAGGGTGAAAAAGGTCCGGAGCACTGGGCGCAGCTGAAGCCGGAATTCTTTTGGTGCAAGCTGAAAAACCAAAGCCCGATCAACATCGACAAGAAGTACAAGGTGAAGGCGAACCTGCCGAAACTGAACCTGTACTATAAGACCGCGAAAGAGAGCGAAGTGGTTAACAACGGCCACACCATCCAGATTAACATCAAAGAGGACAACACCCTGAACTACCTGGGTGAAAAATATCAGCTGAAGCAATTCCACTTTCACACCCCGAGCGAGCACACCATTGAAAAGAAAAGCTACCCGCTGGAGATCCACTTCGTGCACAAAACCGAAGACGGCAAGATTCTGGTGGTTGGCGTTATGGCGAAGCTGGGTAAAACCAACAAGGAGCTGGATAAAATTCTGAACGTGGCGCCGGCGGAGGAAGGCGAAAAAATTCTGGACAAGAACCTGAACCTGAACAACCTGATCCCGAAGGATAAACGTTACATGACCTATAGCGGTAGCCTGACCACCCCGCCGTGCACCGAGGGTGTGCGTTGGATTGTTCTGAAGAAACCGATTAGCATCAGCAAGCAGCAACTGGAGAAGCTGAAAAGCGTGATGGTTAACCCGAACAACCGTCCGGTTCAGGAAATCAACAGCCGTTGGATCATTGAGGGCTTCGAAAACCTGTATTTTCAAGGT,

End with: ctcgag

**SEQ ID: CA6 for *E. coli***

**SEQ name: SazCAtrunc-TEV**

TYPE: PRT; DNA

**PRT sequence** (239 aa; pI, 7.92; MW, 27.7 kDa):

**>CA6-**SazCAtrunc

VHHWSYEGENGPENWAKLNPEYFWCNLKNQSPVDISDNYKVHAKLEKLHINYNKAVNPEIVNNGHTIQVNVLEDFKLNIKGKEYHLKQFHFHAPSEHTVNGKYYPLEMHLVHKDKDGNIAVIGVFFKEGKANPELDKVFKNALKEEGSKVFDGSININALLPPVKNYYTYSGSLTTPPCTEGVLWIVLKQPITASKQQIELFKSIMKHNNNRPTQPINSRYILESNENLYFQGHHHHHH

**DNA sequence** (713 bp; used to generate plasmid Gene6-pET-28b-SazCA-TEV-HisTag):

Start with: ccatgggc

GTGCACCACTGGAGCTACGAGGGTGAAAACGGCCCGGAGAACTGGGCGAAGCTGAACCCGGAATATTTCTGGTGCAACCTGAAAAACCAGAGCCCGGTGGACATCAGCGATAACTACAAGGTTCACGCGAAGCTGGAGAAACTGCACATTAACTATAACAAAGCGGTGAACCCGGAAATCGTTAACAACGGCCACACCATTCAGGTGAACGTTCTGGAGGACTTTAAGCTGAACATCAAGGGTAAAGAATACCACCTGAAACAATTCCACTTTCATGCGCCGAGCGAGCACACCGTGAACGGCAAGTACTATCCGCTGGAAATGCACCTGGTTCACAAGGACAAAGATGGTAACATCGCGGTGATTGGCGTTTTCTTTAAAGAGGGTAAAGCGAACCCGGAACTGGACAAGGTGTTCAAAAACGCGCTGAAGGAAGAGGGTAGCAAAGTTTTTGATGGTAGCATCAACATTAACGCGCTGCTGCCGCCGGTTAAAAACTACTATACCTATAGCGGTAGCCTGACCACCCCGCCGTGCACCGAGGGTGTGCTGTGGATCGTTCTGAAGCAACCGATTACCGCGAGCAAACAGCAAATCGAACTGTTCAAGAGCATTATGAAACACAACAACAACCGTCCGACCCAGCCGATCAACAGCCGTTACATTCTGGAGAGCAACGAAAACCTGTATTTTCAAGGT,

End with: ctcgag

**SEQ ID: CA7 for *E. coli***

**SEQ name: PabCA-TEV**

TYPE: PRT; DNA

**PRT sequence** (185 aa; pI, 6.36; MW, 20.4 kDa):

>CA7-**PabCA**

VVYELNGKKPKIHPSAFIDESAVVIGDVVLEEKTSVWPSAVLRGDIERIYVGKYSNVQDNVSIHTSHGYPTEIGEYVTIGHNAVVHGAKIGNYVIIGIGSVILDGAKIGDHVIIGAGAVVPPNKEIPDYSLVLGVPGKVVRQLTEEEIEWTKKNAEIYVELAEKHLKGRRKLENLYFQGHHHHHH

**DNA sequence** (551 bp; used to generate plasmid Gene7-pET-28b-PabCA-TEV-HisTag):

Start with: ccatgggc

GTGGTTTACGAACTGAACGGCAAGAAACCGAAGATCCACCCGAGCGCGTTCATTGACGAGAGCGCGGTGGTTATTGGTGATGTGGTTCTGGAGGAAAAAACCAGCGTGTGGCCGAGCGCGGTTCTGCGTGGCGACATCGAACGTATTTACGTGGGTAAATATAGCAACGTGCAGGATAACGTTAGCATCCACACCAGCCACGGTTACCCGACCGAAATCGGCGAGTATGTTACCATTGGTCACAACGCGGTGGTTCACGGTGCGAAGATCGGCAACTACGTGATCATTGGTATCGGCAGCGTTATTCTGGACGGTGCGAAAATTGGCGATCACGTGATCATTGGTGCGGGTGCGGTGGTTCCGCCGAACAAGGAAATCCCGGACTATAGCCTGGTGCTGGGTGTTCCGGGCAAAGTGGTTCGTCAGCTGACCGAGGAAGAGATCGAATGGACCAAGAAAAACGCGGAGATTTACGTTGAACTGGCGGAGAAGCACCTGAAAGGCCGTCGTAAACTGGAGAACCTGTATTTTCAAGGT,

End with: ctcgag

**SEQ ID: CA8 for *E. coli***

**SEQ name: PhoCA-TEV**

TYPE: PRT; DNA

**PRT sequence** (185 aa; pI, 6.22; MW, 20.5 kDa):

**>CA8-PhoCA**

AIYEINGKKPRIHPSAFVDENAVVIGDVVLEEKTSVWPSAVLRGDIEQIYVGKYSNVQDNVSIHTSHGYPTEIGEYVTIGHNAMVHGAKVGNYVIIGISSVILDGAKIGDHVIIGAGAVVPPNKEIPDYSLVLGVPGKVVRQLTEEEIEWTKKNAEIYVELAEKHIKGRKRIENLYFQGHHHHHH

**>CA8 DNA sequence** (551 bp; used to generate plasmid Gene8-pET-28b-PhoCA-TEV-HisTag):

Start with: ccatgggc

GCGATCTACGAAATTAACGGCAAGAAACCGCGTATCCACCCGAGCGCGTTCGTGGACGAGAACGCGGTGGTTATTGGTGATGTGGTTCTGGAGGAAAAAACCAGCGTGTGGCCGAGCGCGGTTCTGCGTGGCGACATCGAACAGATTTACGTTGGCAAGTATAGCAACGTGCAAGATAACGTTAGCATCCACACCAGCCACGGTTACCCGACCGAAATCGGCGAGTATGTGACCATTGGTCACAACGCGATGGTTCACGGTGCGAAAGTGGGCAACTACGTTATCATTGGCATCAGCAGCGTGATTCTGGACGGTGCGAAGATCGGCGATCACGTTATCATTGGTGCGGGCGCGGTGGTTCCGCCGAACAAAGAAATTCCGGACTATAGCCTGGTGCTGGGTGTTCCGGGCAAGGTGGTTCGTCAGCTGACCGAGGAAGAGATCGAATGGACCAAGAAAAACGCGGAGATTTACGTGGAACTGGCGGAGAAGCACATCAAAGGCCGTAAGCGTATTGAGAACCTGTATTTTCAAGGT,

End with: ctcgag
